# Evaluation of the Implementation of a Mobile Health App to Support Dutch Primary Care for Diabetes: Qualitative Study

**DOI:** 10.2196/54431

**Published:** 2026-02-09

**Authors:** Liselot N van den Berg, Lisenka te Lindert, Jiska J Aardoom, Anke Versluis, Sofie H Willems, Niels H Chavannes, Marise J Kasteleyn

**Affiliations:** 1 Department of Public Health and Primary Care Leiden University Medical Center Leiden The Netherlands; 2 National eHealth Living Lab Leiden The Netherlands

**Keywords:** diabetes care, implementation, eHealth, lifestyle, primary care, evaluation, mobile health, mHealth, diabetes, qualitative study, Dutch, type 2 diabetes, MiGuide, framework method, quality of life, barriers, facilitators, context-specific, quantitative data, patients

## Abstract

**Background:**

Over 1 million Dutch people have diabetes, of whom 90% have type 2 diabetes. Studies show that lifestyle plays an important role in the course of type 2 diabetes. MiGuide (MiGuide Ltd) is an online platform that helps people adopt and sustain lifestyle changes. The platform is integrated into existing diabetes care within primary care. Previous research has shown that implementing new (eHealth) interventions is challenging and may reduce effectiveness. Mapping out the barriers and success factors in the implementation process is essential so that eHealth interventions such as MiGuide can be used effectively in regular health care.

**Objective:**

This study aimed to evaluate the implementation of MiGuide within Dutch primary care.

**Methods:**

A qualitative study design was used, supplemented by quantitative data from patients. Five general practices participated. Three focus groups (FGs; at baseline, after 6 months, and after 12 months) were conducted with 3 general practitioners, 3 FGs with 8 specialized practice nurses (divided into 2 separate groups with 4 participants per group), 2 FGs (at 6 months and after 12 months) with 5 patients, and 2 FGs (at baseline and after 12 months) with 4 stakeholders from the management of the care group. The implementation process was discussed with health care professionals and management, and usage and user-friendliness were discussed with patients. The framework method was used to analyze the data. The following quantitative data were collected: patient characteristics, user data, and questionnaires at baseline and 6 months, assessing quality of life, usability, and diabetes self-care. The quantitative data were examined using exploratory analyses.

**Results:**

Four themes were found in the qualitative data: “innovation,” “capability, motivation, and opportunity,” “processes,” and “setting.” Different factors within these themes played an essential role throughout the implementation process, such as facilities, technical difficulties, motivation, COVID-19, and the work processes. Areas for improvement were also identified. The supplemented quantitative data showed that usability scored below average at 6 months (mean 53.8; SD 9.3; n=8). Participants had a mean score of 0.84 (SD 0.13) on the EuroQoL-5 dimension and 81.9 (SD 13.4) on the EuroQoL visual analogue scale at baseline. Moreover, the average number of days someone exercised was 4.2 (SD 1.7), and the number of days someone ate a generally healthy diet was 5.1 (SD 1.3). Insufficient data on quality of life and diabetes self-care were collected at 6 months and therefore not presented in this study.

**Conclusions:**

Implementation is a complex process with multiple barriers and facilitators. It is essential to explore the use of context-specific strategies that are aligned with the implementation process phase. Further research is needed to evaluate the next version of the MiGuide platform, which is being implemented in another setting with lifestyle coaches.

## Introduction

In 2018, almost 1 million people were registered with type 2 diabetes mellitus (T2DM) in Dutch primary care [1]. This number has more than doubled since 2004 [2]. Approximately 90% of these people receive treatment in primary care [1].

Following Dutch treatment guidelines for standard diabetes care, patients receive an annual consultation with their general practitioner (GP) and a quarterly or biannual consultation with a specialized practice nurse (SPN). The consultations with the SPN include monitoring of weight, systolic blood pressure, hemoglobin A_1c_ levels, foot condition, and the occurrence of hypo- or hyperglycemia. Individuals with T2DM are also advised to lose weight and adopt a healthy lifestyle, such as sufficient physical activity and a healthy diet. Previous studies have shown that lifestyle plays an essential role in the disease progression of people with T2DM [3-8]. For example, increased physical activity, a healthier diet, and weight loss can lead to remission of T2DM [3,5]. According to the guideline, education on a healthier lifestyle should be provided alongside self-management strategies [9].

Self-management, which is the ability to manage symptoms, treatment, physical consequences, psychosocial consequences, and lifestyle changes, is inherent in living with diabetes [10]. The usage of eHealth self-management interventions for T2DM, such as smartphone apps, has demonstrated potential for adopting a healthier lifestyle. This especially applies to apps targeting diet and physical activity, which can improve health outcomes such as hemoglobin A_1c_. Although self-management apps have potential, it is recommended to offer eHealth as a blended care solution [11].

The success of self-management interventions regarding lifestyle and diabetes depends on the fidelity of the implementation process [12]. Implementation fidelity pertains to the extent to which an intervention is used as intended. Key elements include adherence, intervention complexity, facilitation strategies, quality of delivery, and participant responsiveness [13]. By evaluating whether an intervention has been implemented with fidelity, researchers and practitioners can better understand how and why an intervention works and the extent to which outcomes can be improved. This is crucial because there is often a gap between theory and practice in eHealth implementation. Often, eHealth solutions do not make it to practical use, and when they do, the adoption process is slower than anticipated due to various complex, multilevel challenges [14-17]. For instance, the patient, health care, and organizational levels should be considered [17].

For this study, we followed the implementation of MiGuide (MiGuide Ltd) in primary care. MiGuide is implemented as an integrated part of diabetes care and consists of an app and an online portal. The app allows individuals to track their food intake, physical activity, and specific goals. Lifestyle challenges, such as completing exercise goals each day and consultation preparation, are also available. Measurements, such as blood pressure and weight, are uploaded to the portal, which is accessible to GPs and SPNs. The content of MiGuide is based on the Dutch professional guideline for standard diabetes primary care. This study aims to examine the implementation of MiGuide in Dutch primary care. Our objectives are to identify the barriers and facilitators to the implementation of MiGuide in Dutch primary care and to evaluate the usage of MiGuide.

## Methods

### Setting

This study was conducted among Dutch general practices affiliated with the care group named the GP team (in Dutch: “Het Huisartsenteam” [HHT]) located in the province of Noord-Brabant. A care group is a partnership of several general practices to support logistics and the quality of care [12,18]. The care group HHT cooperated with MiGuide Ltd in an implementation project for the platform MiGuide. Five practices, for which it was technically feasible to link with the MiGuide portal, implemented MiGuide as part of their usual diabetes care. A management team with implementation experts guided the implementation process. It consisted of developers from MiGuide Ltd, eHealth implementation advisors from an external party, and the care group’s board members. MiGuide Ltd provided all instruction materials, provided the experts for training the GPs and SPNs, and was on standby for problem-solving and support. The care group had an intermediary role and informed the general practices.

### Study Design

This was a descriptive qualitative study, supplemented by quantitative data from individuals with T2DM using MiGuide. The study was conducted in Dutch primary care between October 2020 and August 2022.

**Figure 1:**
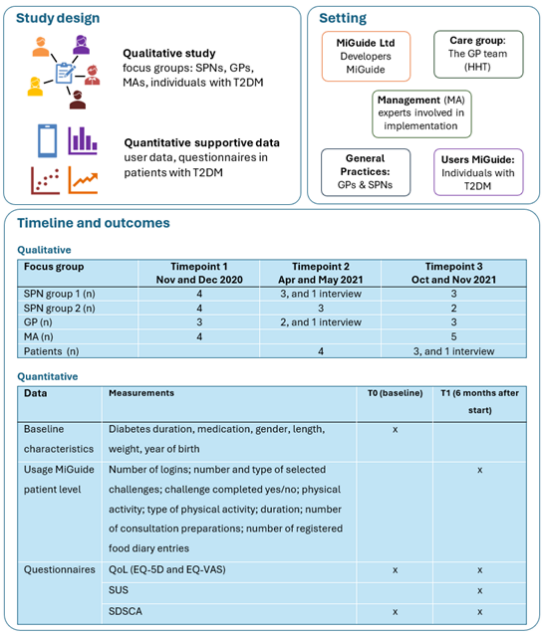
Overview of the study design, setting, timeline, and outcomes. EQ-5D: EuroQoL-5 dimension; EQ-VAS: EuroQoL visual analogue scale; GP: general practitioner; HHT: the GP team (in Dutch: Het Huisartsenteam); MA: management; QoL: quality of life; SDSCA: Summary of Diabetes Self-Care Activities; SPN: specialized practice nurse; SUS: System Usability Scale; T2DM: type 2 diabetes mellitus.

The “implementation fidelity model” [13] was used to examine whether the intervention was used as intended. To evaluate the implementation process, the following aspects were examined: barriers and facilitators, the impact on care pathways, including the transition of care from practice to home, and the role and experiences of GPs and SPNs. We assessed the implementation process at three levels: (1) the practice level from the perspective of GPs and SPNs, (2) the management level from the perspective of experts from the care group and MiGuide Ltd involved in the implementation process (ie MAs), and (3) the user level from the perspective of patients. Quantitative data were used to assess patients’ objective usage of MiGuide, their perceived system usability, and the impact on quality of life and self-care. Figure 1 gives an overview of the design, setting, and outcomes.

### Study Population and Procedure

Patients were eligible to participate in the quantitative study if they (1) were diagnosed with T2DM, (2) were able to read and communicate in Dutch, (3) were treated in a general practice within the care group, (4) had a smartphone (Android version 6.0 or higher [Google], or iOS version 11.0 or higher [Apple Inc]) with access to data connectivity, and (5) were not being treated in a hospital. During the initial phase of MiGuide’s release, one of the exclusion criteria was the participant’s unwillingness to share their data with their health care provider. This criterion was dropped when the second release of the app was launched in July 2021. The inclusion criteria for GPs and SPNs in the qualitative study were (1) being affiliated with the care group and (2) working with MiGuide. Patients also needed to use MiGuide to participate in the focus groups (FGs).

The general practices sent an invitation letter to eligible patients describing MiGuide and the study. Patients could download the app from the App Store or the Google Play Store. At onboarding, individuals indicated whether they agreed to share (1) their user data with their general practice and (2) their email address for research participation. MiGuide could still be used when an individual did not agree. Next, MiGuide Ltd asked for consent via email. Before the second release, users could only use MiGuide when they agreed to share their data for research purposes. As this was not a voluntary choice nor a valid consent, all participants who started using MiGuide before the second release were actively approached to ask for their participation in this study. At the end of the onboarding, patients could visit their GP to verify their identity and receive a unique QR code, but this was not mandatory to use MiGuide. The QR code facilitated the data connection between MiGuide, the health care professional (HCP), and the patient’s medical data. No reimbursement was given for research participation.

For the qualitative part of the study, the care group invited SPNs, GPs, and MAs via email. Patients already using MiGuide were invited to participate via newsletters sent by MiGuide Ltd. All FG members were asked to consent to FG participation via email by the study team. After participation, they received a book about lifestyle and a reimbursement from MiGuide Ltd.

### MiGuide Platform

**Figure 2:**
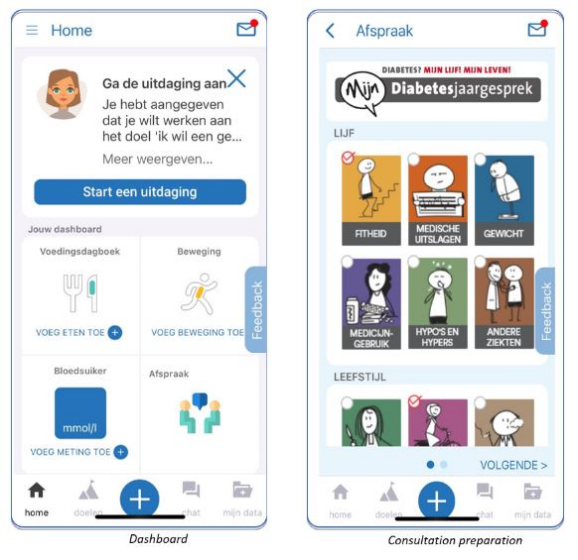
Components of MiGuide.

MiGuide Ltd developed a platform consisting of an app and an online portal to guide individuals with T2DM in changing their behavior to adopt a healthier lifestyle (refer to Figure 2). The content is based on the content of consultations with GPs and SPNs (as described in Dutch treatment guidelines for diabetes in primary care).

The components of MiGuide are organized into the following categories: food diary, physical activity diary, goal setting, and data. The food diary has been improved since the second release by connecting it to an existing app from the Netherlands Nutrition Centre. The physical activity diary offers the possibility to connect with Google Fit. Goal setting offers different goals to enhance control of the following diabetes determinants: blood sugar, blood pressure, sufficient physical activity, prevention or minimization of complications, cholesterol levels, healthy eating and drinking, and healthy weight. The data category comprises the possibility to record blood pressure, blood glucose, weight, and consultation preparation. This data can be shared with the information system of the user’s general practice if the user agrees to do so. The consultation preparation component allows users to send notes to their general practice about themes they want to discuss during a consultation with their GP or SPN. The themes they can choose from consist of physique, lifestyle, well-being, and self-care.

### Data Collection

#### Qualitative Study

FGs and interviews were conducted using video conferencing platforms (Microsoft Teams or Jitsi). The FGs and interviews were audio-recorded.

FGs with GPs, SPNs, and MAs took place (1) during the initial adoption phase, (2) at 3 months after the implementation, and (3) at 12 months after the implementation (end of the study period). The FGs with individuals with T2DM took place (1) 3 months after initiating the use of MiGuide and (2) at the end of the study period.

To reach data saturation, we aimed for 5 GPs, 10 SPNs (divided into 2 groups), 4 MAs, and 4 patients. In cases where it was not possible to gather all members of an FG simultaneously, separate interviews were conducted to collect additional data.

#### Quantitative Study

Figure 1 provides the usage data of MiGuide, along with the questionnaire data collected at the user level. The usage data were gathered through an automated process. Users received in-app notifications at baseline and at 6 months to complete the questionnaires, with demographic characteristics collected only at baseline. Subsequently, MiGuide Ltd sent manual email reminders when participants had not completed the questionnaires.

### Analysis

#### Qualitative Study

A thematic analysis based on the framework method procedure [19] was performed. At stage 1, the audio of all interviews was automatically converted to transcripts and pseudonymized. Two researchers (LvdB and LtL) became familiar with the interviews (stage 2) by checking and supplementing the transcription and making notes and summaries of the interviews. At stage 3, the researchers LvdB and LtL started coding the first transcripts using a mainly deductive approach. A concept codebook was created before coding, based on the implementation fidelity model and the above-mentioned aspects to evaluate the implementation process. The first 2 FGs with SPNs and the first of the other predefined FGs were coded independently by LvdB and LtL. At stage 4, the analytical framework (codebook) was further developed by comparing the independently coded FGs and agreeing on the definition of codes and categories by LvdB and LtL. If applicable, new codes were added to the codebook. The codes and categories were then applied to the remaining transcripts at stage 5. These transcripts were divided by LvdB and LtL and coded by one researcher. At stage 6, the data were charted into the framework matrix. Data from each transcript were summarized and provided with illustrative quotes. Each predefined FG was treated as a different unit of analysis, thereby assessing the implementation process at different levels: patient, GP, SPN, and MA. Finally, at stage 7, characteristics and differences between the data were identified. This process was supervised by senior researchers MJK and SHW.

#### Quantitative Study

Normally distributed numerical data were described as mean (SD). Nonnormally distributed numerical data were described as median with IQR. Categorical data were described as frequencies with percentages. Descriptive statistics were performed for the collected quantitative data using IBM SPSS Statistics (version 25) [20].

### Ethical Considerations

The study did not fall within the scope of the Dutch Medical Research Involving Human Subjects Act, according to the Medical Research and Ethics Committee of VU Amsterdam Medical Center (2020.0708). Subsequently, a declaration of no objection was obtained from the Medical Research and Ethics Committee. Participants provided informed consent and were able to opt out from the study (refer to the “Procedure” section). Both quantitative and qualitative data were collected pseudonymously.

## Results

### Demographic Characteristics

In the qualitative study, 3 patients were male (3/5, 60%), all 9 SPNs were female (9/9, 100%), all 3 GPs (3/3, 100%) were male, and 5 MAs were male (5/6, 83%).

In the quantitative study, 58 participants were enrolled. Half were male (29/58, 50%), the mean age was 60.1 (SD 12.1), the median BMI was 30.3 (IQR 25.9-35.8), and 19% (11/58) of the participants were paired with the MiGuide portal. The demographic characteristics and number of paired patients per participating practice are provided in Table 1.

**Table 1:** Baseline data of participating practices.

Characteristic	Practice 1 (n=4)	Practice 2 (n=42)	Practice 3 (n=7)	Practice 4 (n=5)	Total (N=58)
Paired patients, n (%)	0 (0)	8 (19)	2 (28.6)	1 (20)	11 (19)
Age (years), mean (SD)	60.6 (7.0)	61.6 (11.3)	50.5 (11.7)	60.7 (19.3)	60.1 (12.1)
Male, n (%)	2 (50)	23 (56.1)	1 (14.3)	2 (50)	29 (50)
BMI^a^, median (IQR)	27.9 (24.8-32.9)	30.3 (26.8-36.4)	32.3 (28.7-35.8)	25.7 (22.6-40.3)	30.3 (25.9-35.8)

^a^BMI data were complete for all participants except one individual in practice 2.

### Quality of Life and Self-Care Baseline

At baseline, the mean EuroQoL-5-dimension score was 0.84 (SD 0.13), and the mean EuroQoL visual analogue scale score was 81.9 (SD 13.4). The mean number of days participants exercised was 4.2 (SD 1.7), and the mean number of days they followed a generally healthy diet was 5.1 (SD 1.3; refer to Table 2). Insufficient data were collected at 6 months (by 3, 4, and 6 participants, respectively); therefore, these data are not presented.

**Table 2:** Quality of life and self-care scores at baseline.

Questionnaire			Baseline value
EQ-5D (n=21), mean (SD)			0.84 (0.13)
EQ-VAS^a^ (n=16), mean (SD)			81.9 (13.4)
**SDSCA^b^ (n=13), mean (SD)**			
	No of days of physical activity or exercise	4.2 (1.7)	
	No of days of glucose monitoring	1.4 (2.0)	
	No of days of foot care	1.5 (1.7)	
	No of days of a healthy diet (general)	5.1 (1.3)	
	No of days of a healthy diet (specific)	5.3 (0.9)	
Nonsmoking, n/N (%)			9/11 (81.8)

^a^EQ-VAS: EuroQoL visual analogue scale.

^b^SDSCA: Summary of Diabetes Self-Care Activities.

### Innovation

Patients were enthusiastic about the MiGuide concept but mentioned that the app was not intuitive for navigation. Later, during FG2, they reported that the app’s design improved and looked clean and clear. They often used the MiGuide app daily, ranging from 5 to 60 minutes.

The 2 most frequently used functions were the food diary and physical activity tracking. Nevertheless, patients thought the food diary was too generic. They were also critical of the physical activity component: the point system was difficult to interpret. Some used pedometers and wondered why their results were not directly visible in the app. MiGuide Ltd immediately processed the food diary feedback after FG1, resulting in more enthusiastic patients during FG2. They were also positive regarding plans to change the physical activity functionality at a later stage. In the quantitative study, MiGuide usability at 6 months had a mean score of 53.8 (SD 9.3; n=8; range 20-90), which was below average.

Patients indicated that they rarely used consultation preparation; some used challenges, goals, and daily tips. This was also seen in the quantitative data (refer to Figures S1-S3 in Multimedia Appendix 1). During the FGs, patients stated that consultation preparation was not used because they thought it was not very useful for the SPN or themselves. For example, measurements were already available for SPNs, or patients wanted to discuss their T2DM during a consultation. They also mentioned that they already used the care group’s app (ie, the HHT [Pharmeon BV] app) to access blood results and visits.

All patients, except one, said they expected to keep using MiGuide long-term. They expected a positive course of their T2DM while using MiGuide because it could motivate them to increase physical activity and eat healthier. Greater insight allowed them to make more impactful lifestyle changes. During FG2, they stated that they became more aware of their T2DM and that the app provided insight into their lifestyle.

For me, the app works very well. I mean, if I decide that day to misbehave and drink some craft beers, delicious, on Saturday night. Then I will put it in the app and think, “yes, this was not the best for me, but it was delicious.” I think if you improve the app further, you will become aware of yourself. I think that is the most important part of the app. This is the reason I fill it in loyally.

PT3 during FG1; April 22, 2021

I have to admit, I never thought I would when they told me to lose 30 kilos first… At the time, I did not think it was a dot on the horizon; it was a dot behind the dot. And afterwards, with some attention, you can lose 20 kilos quite easily.

PT3 during FG2; September 23, 2021

SPNs and GPs mentioned that the MiGuide platform has potential, may be insightful, and looks nice. Initially, navigation on the platform was suboptimal. SPNs mentioned during all FGs that they were not working much with MiGuide and were not actively promoting it in their practices (refer to “Processes” and “Setting” sections). The GPs stated that mainly the SPNs worked with the app. They both experienced a low number of users (2-5 patients using the app per practice); while downloads increased over the year, SPNs indicated that patients stopped using the app, mainly due to the large number of notifications. One GP also said that patients stated that they were already using the HHT app or another app that helps with lifestyle changes and T2DM. The SPNs and GPs noticed that consultation preparation was not being used. Moreover, patients decided which features they wanted to use in the app without the guidance of the SPNs.

One of my SPNs has also downloaded the app herself and also tries to fill in things herself, but it does not seem to be very user-friendly. For example, with filling in food: if you have eaten salmon, for example, you have to click a lot to fill in the right amount of what you have eaten. The moment you have entered certain goals and you do not do it, you receive messages at the most impossible times that you have not achieved it or have not done it yet. This then causes people to say, “I do not need to get messages like that at midnight.” Then they are done with it very quickly.

GP1 during FG2; April 15, 2021

At all 3 time points, the SPNs and GPs reported technical difficulties, mainly in linking the app with their information system via the portal and the QR code. The question arose among SPNs during FG1 if the connection with the information system was necessary and would add value. One of the GPs stated that they were happy that MiGuide was not directly linked to the information system and that they could find the data in a separate section instead of directly in the PT’s record.

The problem with the fact that you cannot send via a safe environment is, of course, a pity. The link has not yet been made with [name of the information system]. Yeah, otherwise, it did not work out this week, so I have a hotline with [name of MiGuide employee], and they are resetting it, and yet, it is not running yet.

SPN4 during FG1; November 10, 2020

During FG1, the SPNs were asked about their expectations of MiGuide on PTs’ lifestyle and self-management. They believed the app would enable better monitoring of individuals with T2DM and facilitate exchanging information. They found it useful for patients with an affinity for eHealth, and the app could provide patients with valuable insights into their lifestyle. However, there was some concern about whether the app would meet the needs of patients and whether they would view it as a self-management tool or would instead rely heavily on the SPNs for support. One of the GPs also stated that it is a great app for patients, with many interesting features, and that it would be better to focus on the self-management part of the app.

If PTs enjoy using such an app, I would like to encourage them to do so. Because I think it is a great app for PTs in itself, with a lot of interesting things in it, such as lifestyle advice and things like that. […] I think those things that the PT can do themselves that they are good. That is where I would focus. So, then it is a self-management app.

GP2 during FG3; November 9, 2021

During FG1, someone from MiGuide Ltd explained that MiGuide aimed to improve the patients' self-management. The patients could get more insight into their disease, improve their lifestyle using MiGuide, and hopefully have another type of conversation with their HCP due to the use of the consultation preparation function. A care group (HHT) representative emphasized that MiGuide aligned perfectly with their 5-year plan to prioritize the creation of a continuum between the patient and practice and the establishment of a connection between HCPs and patients, thereby providing blended care.

Which, of course, we see in recent years, that piece of lifestyle medicine, that it is increasingly well substantiated and that it really will become the leading way to apply it. At the same time, it is also complex to implement and introduce. So, it is not just the chronic disease itself, but also the rise of lifestyle medicine where we will find substance.

MG1 during FG1; December 2, 2020

During FG1, the MAs discussed that all patients should be invited to use MiGuide instead of inviting based on assumptions (eg, not asking because of the patient’s age). However, accepting that the app is not for everyone is essential. HCPs could become frustrated if they aim to reach 100% of patients. The first 10%-20% could be easily reached; thus, HCPs should focus on this group. Knowing the benefits of using the platform could make it more appealing to those not initially enthusiastic about it. It may not work to convince individuals already resistant to new technology. Instead, a more promising approach may be to target individuals who already do a lot online (eg, planning e-consultations).

So yes, ask everyone, but also accept the no. There is already enough extra energy to get this off the ground, but do not try to pull the person up to their knees in the mud. They will follow. Focus on the group that wants it now and is also aware that it is not a solution for everyone, so the “digital unless” is also one that often passes by; there are always patients who are not language-skilled enough or who do not master this part. Accept that too, because if you think you have to achieve 100 percent, you will only frustrate yourself.

CZ1 during FG1; December 2, 2020

During FG2, the MAs discussed the requirements to initiate changes in the process at the patient level. A transition is needed for patients to have autonomy over their health. Concerns remained about whether the process was clearly defined for the SPN and the patient and whether they could check the progress of their goals. Someone from HHT wondered whether the MiGuide platform could create an extra burden for SPNs.

The MAs envisioned a transition toward increased self-management for the patients, less workload in the practices, and eventually a decrease in the number of consultations.

### Capability, Motivation, and Opportunity

The SPN or GP informed the patients about MiGuide and invited them to use it. During FG1, patients mentioned that they had not yet paired MiGuide with the QR code. One patient was wondering whether the connection would be acceptable to the GPs, as they had a high workload. However, the enthusiasm from their HCP encouraged the patients to use the app.

One patient reported difficulties logging into the app; 9 out of 10 times, they needed to start the app twice before logging in. This problem emerged a couple of weeks (2 or 3 weeks) before FG2 and resulted in less motivation to use it. Due to the availability and use of the HHT app, some patients explained that they did not need MiGuide (refer to the “Innovation” section).

The patients expressed that it is a matter of self-discipline, structure, and persistence to use or keep using the app.

I think the fact that you have diabetes should be enough. I think the motivation to fill it in should come from within yourself. If it does not come from yourself, then I do not think you will get it done.

PT3 during FG1; April 22, 2021

The enthusiasm of both patients and MiGuide Ltd stimulated the SPNs to work with MiGuide. The importance of enthusiasm from others was repeated throughout all FGs. The SPNs stated during the last FG that it would motivate and activate their enthusiasm if they were reminded by personal visits instead of only reminder emails from MiGuide Ltd or the care group.

In our role, we are really like, “Oh, that is new, and we want that, and we are going to work on that.” Well, yes, SPN7 and I, if I speak for the two of us, we are also really always at the forefront of rolling out new things, implementing and informing patients about that, and collaborating…

SPN9 during FG1; December 10, 2020

During FG1 and FG2, SPNs expressed that they had little experience with MiGuide and needed more knowledge before working with the platform. They agreed that extra training was necessary and that more experience would help them use MiGuide in the long run. One GP stated that it should become completely “dummy-proof.” MiGuide Ltd appreciated this feedback and organized extra demo sessions and masterclasses for HCPs to learn about the platform. The demo sessions helped motivate and activate the SPNs, as they became more confident in informing patients. Only some GPs followed the masterclass and demo session, but they were satisfied with the information they received.

During the initial implementation of MiGuide, SPNs mentioned that GPs did their best to be involved and to stimulate the use of MiGuide. However, over time, GP involvement decreased, and the SPNs felt less supported. They also mentioned that they did not experience much facilitation and support from the care group (refer to the “Processes” section). On the other hand, MiGuide Ltd and its helpdesk were accessible and helpful, which was valued.

Moreover, the FGs helped motivate SPNs by exchanging thoughts about MiGuide. The SPNs and GPs stated that the motivation and enthusiasm of HCPs were essential facilitators. Many HCPs were skeptical, leading to friction when patients wanted to use the app. It would help to see why the innovation is valuable (eg, a decrease in the workload). The added value could also be used in other practices to motivate them to implement the platform.

You know, if I tell a patient that they can come twice a year, I do not want to have to answer 80 more messages in those six months, because then I would rather have that they just come every three months, instead of twice a year, if I need to have so much other contact with the patient.

SPN5 during FG3; October 6, 2021

A participant from HHT explained that the 5 practices were informed by MiGuide Ltd, and documentation was prepared for these practices (eg, a manual and webinar). The initial release date was delayed due to technical issues (refer to the “Innovation” section). In the starting phase, the care group frequently contacted the first 2 practices and MiGuide Ltd about the implementation, which was acknowledged as a facilitator. During FG2, a participant from MiGuide Ltd stated that contact with the practices decreased while communication seemed essential. Nonetheless, practices were able throughout the implementation phase to contact MiGuide Ltd.

During FG2, the MAs also noticed resistance from the HCPs to use MiGuide as a standard method. SPNs and GPs felt that the benefit was too minimal and not immediately noticeable (it cost a lot of energy or time, and they did not immediately gain something in return). The MAs experienced the same issues while changing telephone appointments to digital (online) appointments.

That is true, and I am certainly not going to argue that online appointments are an advantage for the assistants. But make no mistake that they spent quite a while sabotaging you to prevent people from making online appointments. And why? Because that effect was not immediately apparent. They thought only when they experienced the effect, “We have to start enthusing about it.” But before that, we were almost literally on top of it, just to keep saying, “No, do it now, do it now. The benefit will come to use later.” That it will come later, that is just tricky.

HHT2 during FG2; October 14, 2021

Funding was an essential facilitator for implementing and integrating MiGuide into the practices. However, this could be a problem in the long term because there is no structural funding yet. The MAs also noticed that the FGs resulted in increased motivation among the SPNs to use MiGuide.

### Processes

The SPNs expressed in FG1 and FG3 that the implementation of MiGuide was imposed upon them by HHT (ie, in a top-down manner). They indicated that the implementation process could have been improved if they had been allowed to provide input. The GPs also acknowledged these issues concerning the implementation within their practices. The top-down approach, along with other factors outlined under “setting,” resulted in MiGuide having lower priority for both SPNs and GPs.

I think HHT wants to be upfront and do new things and try new things, and that is also a lot of fun. And sometimes you think, “Yes, it is already completely thought through in all layers,” because the bosses behind their desks say, “Okay, we are going to do this, and MiGuide and is fun; we give it a chance.” And eventually, we are the ones who need to do it. Yes, and then the question is whether it is thought through, like, do the SPNs have time? How should they do it? Do they have enough training, support, etc.? Yes, it sometimes is; yes, maybe not high enough on the list, I think.

SPN7 during FG3; October 13, 2021

As mentioned by GPs and MAs under the “Capability, Motivation, and Opportunity” subheading, it would motivate HCPs to know that the implementation of MiGuide would help reduce their workload in the longer term. SPNs were aware that there would initially be an increased workload due to the changes in their work processes (eg, becoming accustomed to the app and preparation time). During FG3, SPNs anticipated that the workload would further increase due to the potential for patients to ask questions via the app and the need to personalize the program for all users. However, they reported no differences in MiGuide use on daily care, workload, and job satisfaction since the previous FG. On the other hand, GPs expected during FG1 that the function of consultation preparation could positively influence SPNs’ consultations because of the possibility of low-key communication.

With the daily process, not a lot, I would say. With diabetes, I have an average of 100; thus, then we still have over 2000 patients. So yes, I think it is a bit, as I stated earlier. I hope that patients are motivated to do more with their lifestyle and that they will receive feedback that it will work. And I hope, on the other hand, that the health care professional gets something out of it because patients will get to the consultation better prepared. That you have already answered their questions for an important part. Yes, and I hope that the app’s usage over a longer period can replace the number of consultations. That these can be dropped.

GP2 during FG1; November 17, 2020

I had one patient who did prepare, but only 20 minutes before their appointment. They stated, “Oh yes, I forgot, but I need to do this for you.” Yes, then I think, you do not need to do it for me; you should do it for yourself. And what they select, I cannot work with that, because this patient has a lot of mental issues, and they want to discuss this during the consultation. Yes, I cannot help them with that.

SPN5 during FG3; October 6, 2021

During FG1, MAs recognized the need for management to implement an innovation. They play a crucial role in providing necessary support, such as organizing webinars, creating guides, offering workarounds, being available for questions, motivating and stimulating individuals involved in the implementation process, and showing the progress of the implementation. One important aspect highlighted by the participants was that digital projects often differ from initial expectations. However, this difference is not viewed as a problem since continuous development and integration of feedback usually lead to improvements.

One aspect that needs to be considered is that it is difficult during implementation to immediately have a fully operational innovation. Therefore, managing expectations and ensuring clear communication about what aspects work and what aspects do not work is crucial. Failing to address these factors from the beginning could result in disappointment. Providing workarounds (ie, guidelines on what to do when encountering issues with the platform) and offering more intensive implementation coaching could facilitate a smoother transition. The first experience with the platform and the timing of the implementation were also pivotal, and greater emphasis could have been placed on the testing phase.

[…] but there are also a lot of them who take that step with a bit more difficulty than we do, and when they run into things like that, you quickly have an excuse to say, “Hey, wait a minute. That should not be done yet.” That does then cause a delay, and it also ensures that you do not yet have the results, as you just asked for the study OZ1. If it is all there, and what do we think of it in practice?

CZ1 during FG1; December 2, 2020

A representative from HHT experienced difficulty with promoting MiGuide in the practices since it deviated from the regular work processes. Adopting MiGuide required HCPs to shift from a traditional medicine approach to a lifestyle medicine approach. The question remained as to how MiGuide could be integrated into daily routines. As mentioned under the “Capability, Motivation, and Opportunity” subheading, gaining insight into reports could be essential in demonstrating the results of SPNs’ efforts. Communicating and gathering feedback could contribute to improving the implementation.

### Setting

During all FGs, the SPNs and GPs expressed that there was too much going on in their practices, which negatively affected the implementation of MiGuide. The MiGuide platform was implemented during the COVID-19 pandemic, and this influenced the implementation process (eg, other work processes were needed during the pandemic). Furthermore, other factors in the setting greatly influenced the implementation process of MiGuide: reorganization, absenteeism, turnover, and being too occupied with usual diabetes care (ie, many disrupted patients due to COVID-19).

I have not paired any new people, and I also notice that I have experienced a very hectic summer here in the practice, so I was actually happy to be able to do my daily tasks, and because of that, I have made MiGuide a lower priority. Maybe not the idea, but I was glad that my days were done at some point or that I had done what I had to do, and yes, I found the summer quite tough, let’s put it that way.

SPN5 during FG3; October 6, 2021

During FG1, SPNs stated that it would be easier to implement MiGuide when sitting across patients to show the app. After a year, GPs noticed that patients were coming back to the practice after the COVID-19 pandemic for regular consultations. These consultations could be the perfect opportunity to discuss MiGuide with the patients. They noticed that the strength of the MiGuide platform is the connection with the practice and that MiGuide Ltd could focus more on promoting this aspect (refer to “Innovation” subheading).

The MAs also recognized that the COVID-19 pandemic influenced the implementation process of MiGuide. For example, the explanation about MiGuide was held digitally instead of physically in the practices. This resulted in uncertainties about how the MiGuide platform, especially the app, worked. They also acknowledged other barriers in the setting of the practices: pregnancy leaves, absenteeism, turnover, and multiple programs (eg, Acceleration Program Information Exchange Patient and Professional 5 [in Dutch: Versnellingsprogramma Informatie-uitwisseling Patiënt en Professional 5] and the transition from one information system to another) being implemented and used in the practices.

During FG1, the ecosystem of the care group was mentioned as a great facilitator. The fit between MiGuide and the goals of HHT helped with the commitment to the project. The MAs also noticed that the process could have changed if people had met physically in the practices. It also helps when practices have embraced lifestyle medicine (refer to the “Processes” subheading).

At the beginning, of course, there was also the part that everyone was going to work more from home and more digitally. If that had not been the case, we might have been able to take MiGuide physically into the practices in the beginning, as was initially planned. So, I do not know if that would have changed the process, but that is perhaps still a point of attention.

HHT1 during FG1; December 2, 2020

### Suggestions for Improvement

During the FGs, improvement possibilities regarding the MiGuide app were discussed. In this section, only feedback regarding not yet implemented features or processes is discussed.

#### FGs With Patients

Patients suggested several improvements for the MiGuide app. These included (1) the implementation of speech-to-text functionality, which would not only streamline the app’s usability for the patients themselves but would also be helpful for the older population and individuals with visual impairments; (2) enhanced tracking of physical activity, with a preference for incorporating a pedometer or a calorie-based system for the physical activity feature; (3) the addition of medication tracking, including a medication management feature within the app; (4) a streamlined login procedure to simplify the login process and enhance user experience; and (5) the introduction of engaging physical activity challenges, reflecting a desire for more challenging elements regarding the physical activity feature.

#### FGs With SPNs and GPs

Suggestions regarding the MiGuide platform included (1) reduction of questionnaires to minimize the number of questionnaires for patients; (2) customizable notifications, allowing patients to personalize the frequency of notifications; (3) improved personalization, such as linking automatic questions to specific outcomes—for instance, when an outcome shows an increase, the platform can generate questions regarding potential factors such as medication changes or stress levels, which can then be discussed during consultations between the SPN and patient to identify strategies for improving outcomes; (4) a notification for consultation preparation integrated into the information system to alert HCPs when a patient has prepared their consultation; and (5) automated synchronization of blood pressure measurements to enhance user-friendliness.

Suggestions regarding the implementation of MiGuide included (1) preimplementation testing, in which a willing person could test the app and give feedback on their experience, allowing SPNs to familiarize themselves with the app, gain valuable insights, and ensure a smooth integration into their practices. This test phase also facilitates a foolproof system; (2) streamlining the manual by creating a concise version to enhance clarity and comprehension of MiGuide; (3) facilitating knowledge exchange among SPNs and GPs, including exchanging tips to improve the implementation process, and evaluating the current implementation process with colleagues; (4) involvement of GPs and the care group (HHT) to contribute to smoother adoption and usage of the MiGuide platform, with 2 or 3 responsible individuals designated to oversee implementation to ensure effective coordination and support; (5) involvement of other HCPs, such as physiotherapists or lifestyle coaches, to foster greater engagement and usage of the platform; (6) improving visibility via commercials, such as health insurance campaigns or newsletters from HHT, to encourage patients to use the app; (7) emphasis on report insights and best practices, providing practical examples to offer valuable guidance and facilitate evidence-based decision-making during implementation, while highlighting that workload would decrease after the implementation of MiGuide because this is experienced as the biggest motivator for HCPs to implement and use MiGuide; and (8) integration with the HHT app rather than functioning as a stand-alone tool, ensuring seamless integration into the existing HHT app and prioritizing ease of use for both patients and HCPs.

#### FGs With MAs

Suggestions regarding the MiGuide platform included (1) expansion of the MiGuide platform to include other chronic diseases beyond its current scope, such as CVRM and obesity. Suggestions regarding the implementation of MiGuide included (1) preimplementation planning and testing, in which it is recommended to incorporate a phase for testing and developing a plan or workaround before the actual implementation; this tailored approach ensures that all aspects, including timing, motivation, technical processes, and user-friendliness, are thoroughly addressed to optimize the overall implementation process; (2) improved communication with HCPs and enhanced expectation management, as clear communication and proactive management of expectations will contribute to a smoother adoption and usage of the MiGuide platform, with interventions, comprehensive instructions, gathering feedback, and fostering a collaborative approach helping to improve communication; (3) securing structural funding to ensure the sustainability of the implementation, with efforts directed toward securing funding for ongoing support and maintenance of the MiGuide platform; (4) focus on engaging participating patients, acknowledging that achieving 100% engagement may not be feasible and that the focus should be on the group that is motivated and enthusiastic; (5) emphasis on report insights and best practices, as reports and practical examples offer valuable guidance and facilitate evidence-based decision-making during the implementation process; (6) integration with lifestyle programs or interventions, considering that the MiGuide platform is not yet a part of diabetes care and exploring opportunities to integrate the platform with existing lifestyle programs or interventions to enhance its utility, with integrating a lifestyle coach could further enhance the app’s effectiveness; (7) involvement of other HCPs, such as physiotherapists or lifestyle coaches, who have more frequent interactions with individuals with T2DM and can foster greater engagement and usage of the platform; and (8) acceptance of trial and error, recognizing the iterative nature of the implementation process and being aware of the trial-and-error approach that allows for continuous learning, adjustment, and refinement. 

## Discussion

### Principal Findings

The study examined the implementation of the MiGuide platform within 5 Dutch practices of a care group. During the implementation, it became clear that the adoption phase took longer than expected. The group of users was also lower than anticipated; however, this group was motivated and enthusiastic. When implementing an innovation, such as MiGuide, it is necessary to focus on engaging a small group of enthusiastic participants, continuously learning and reporting insights from this small group via an iterative process, and concentrating beforehand on expectation management. Furthermore, developing clear guidelines for the implementation and a workaround (ie, guidelines on what to do when encountering issues with the platform) and thorough testing of the innovation could contribute to a successful implementation. As discussed with the HCPs and MAs, the innovation should fit within their daily work, and there should be sufficient involvement from all parties within the practice to stimulate the adoption.

The COVID-19 pandemic had a big impact on the implementation of MiGuide. Other studies also examined the impact of COVID-19 on implementation and found that the pandemic resulted in unexpected situations and that the setting in which the innovation was delivered was essential for the success of the implementation [21-23]. This was also the case for MiGuide: an unforeseen transition from a physical implementation process toward a digital implementation process and more disrupted patients due to the pandemic, which increased the workload of HCPs. Therefore, MiGuide had a lower priority within the list of daily tasks of the HCPs.

Compared to the framework for implementation fidelity [13], adherence was relatively low, and therefore, implementation fidelity was relatively low in this study. Most patients used the app every day for 5-60 minutes, which aligned with the recommended “dose” and “duration”; however, the HCPs were still in the start-up phase (eg, sending out invitations) and were not actively promoting MiGuide 1 year after the start of the implementation. The potential moderators in the framework [13] should also be considered. Some participants stated that the platform was easy to use, while others had more difficulties, especially in terms of navigation. The strategies to facilitate the implementation and quality of delivery were relatively high due to the manual, webinar, and accessible support desk of MiGuide Ltd. This could be further improved by creating a workaround and discussing with the practices what their needs are to facilitate fitting strategies. The last moderator, participant responsiveness (eg, enthusiasm), was high among most patients, while it declined over time for HCPs. All these factors may have affected the implementation process of the MiGuide platform.

A scoping review conducted on the implementation of eHealth interventions for hypertension or diabetes [24] revealed similar barriers hindering the adoption. Among these barriers were technical issues, complexities associated with the integration of nonroutine processes, time and attention requirements for usage, centralized decision-making structures, high turnover, low HCP engagement, limited prioritization of the intervention compared to existing initiatives, shortage of funding, and ambiguities surrounding staff responsibilities during implementation. This review also emphasized that the likelihood of successful implementation increased when interventions align with and become an integral part of existing organizational goals and workflows [24]. Other studies also found similar barriers and facilitators and stated that the challenges around implementation are multilevel and complex [14,16,25]. Furthermore, when innovations are adopted, implementation science is not always considered, even though implementation science may ensure that innovations are making sustainable positive differences [26]. To successfully implement an eHealth innovation, such as MiGuide, it is essential to explore the use of context-specific strategies that are aligned with the implementation process phase [15]. MiGuide Ltd considered the importance of the fit of their innovation with current workflows and therefore changed the implementation setting from GPs and SPNs toward lifestyle coaches [27]. The upscaling of MiGuide is currently being supported through two programs: (1) CooL-MiGuide (a lifestyle intervention program reimbursed under the basic health insurance package in the Netherlands) and (2) MiGuide-Direct (a program focusing on the combined app of glucagon-like peptide-1 medication and lifestyle interventions). This could help to scale up the implementation of MiGuide to reach a larger population.

### Strengths and Limitations

This study had several strengths and limitations. An essential strength was the observational setting, which minimized the interference with the dynamics of daily practice and facilitated the inclusion of perspectives of HCPs and patients. A second strength was that MiGuide Ltd gathered the input after each FG, whereby the most frequently discussed suggestions were discussed and, when possible, addressed.

A limitation was that the quantitative data were insufficient for profound analyses due to the low sample size. This was partly due to the required adjustments in the consent procedure and partly due to a representative reflection of the real situation in which data are not easily obtained because of high dropout. Consequently, we were not able to conduct an extensive quantitative analysis as originally planned. It is therefore necessary to carefully interpret the study data due to the limited generalizability of the findings. In addition, it is essential to be aware of potential selection and social desirability bias, which may have occurred during data collection due to the pseudonymous character of this study. Furthermore, as previously discussed, the COVID-19 pandemic had a great impact on the implementation and the study.

### Implications for Future Research and Practice

In this study, a minimal viable product was examined. In a future study, an improved version could be evaluated via a hybrid type II trial [28] with a focus on both implementations, usage, and outcomes. The focus could also be on the integration of the MiGuide platform with a lifestyle program or intervention. It would also be beneficial to include a more diverse range of HCPs, such as lifestyle coaches, and patients from different demographic backgrounds to enhance the generalizability of the findings. This study and the new setting could then be compared regarding barriers and facilitators for the implementation process. Moreover, potential risks—such as an over-reliance on technology or decreased face-to-face interaction between patients and HCPs, and possibilities for mitigating these risks—could be examined. The usage of artificial intelligence could also be a theme to further explore. MiGuide Ltd already incorporates artificial intelligence for two primary purposes: (1) the development of personalized content and (2) the analysis of user data and lifestyle patterns. Further possibilities with artificial intelligence could be explored.

Furthermore, it is essential to acknowledge that an eHealth innovation is a dynamic product that needs to be regularly evaluated and adjusted where necessary. The maintenance of eHealth also needs proper funding, as both are associated with ongoing costs. As stated by MAs and found in previous studies [15,29], not having structural funding is a barrier during the implementation process. Therefore, costs should be considered during further implementation of MiGuide. In a future study, the cost-effectiveness and economic impact of MiGuide, including how potential benefits may be distributed across stakeholders, such as patients and HCPs, could be assessed. A comprehensive cost-effectiveness analysis could also inform evidence-based policy decisions and support the development of sustainable reimbursement models for the broader implementation of digital health interventions such as MiGuide.

In addition, a designated implementation team could improve the success rate of the implementation. For example, a previous study found that implementation efforts with designated teams led to an 80% success rate versus 14% during implementation efforts that did not have such teams [30]. Although MiGuide Ltd gathered a team of experts in implementation, this could be further enhanced throughout the process and adjusted to the implementation phase.

### Conclusions

This study evaluated the implementation of MiGuide, an eHealth innovation that promotes a healthier lifestyle among individuals with T2DM, within primary care. The implementation is a complex process, and barriers, also found in previous studies, such as technical issues and time and attention requirements for usage, were established by the participants. Essential factors for a successful implementation are engaging a small group of enthusiastic participants, continuously learning and reporting insights, and concentrating beforehand on expectation management and testing of the app. In addition, it is important to explore the use of context-specific strategies that are aligned with the implementation process phase. Further research is needed to evaluate the new setting with lifestyle coaches in which an improved platform is integrated.

## Appendix

Multimedia Appendix 1 User data MiGuide.

## Abbreviations

FG: focus group
GP: general practitioner
HCP: health care professional
HHT: Het Huisartsenteam
MA: management
SPN: specialized practice nurse
T2DM: type 2 diabetes mellitus
