# Supplementary material for: Evaluation of the Implementation of a Mobile Health App to Support Dutch Primary Care for Diabetes: Qualitative Study
Source: JMIR Hum Factors. 2026 Feb 9;13:e54431. doi: 10.2196/54431 (PMC12930143; doi:10.2196/54431)
Supplement: Multimedia Appendix 1 [file humanfactors_v13i1e54431_app1.docx]

**Figure S1.** Number of goals and challenges selected by participants.

**Figure S2.** Number of measurements (eg, blood pressure) and activities (eg, physical activity).

**Figure S3.** Number of food diary registries, including the old diary (before and during FG1 with PTs) and the new diary (before and during FG2 with PTs).
